# Stickler Syndrome: Airway Complications in a Case Series of 502 Patients

**DOI:** 10.1213/ANE.0000000000004582

**Published:** 2019-12-16

**Authors:** Julia Zimmermann, Daniel J. Stubbs, Allan J. Richards, Philip Alexander, Annie M. McNinch, Basil Matta, Martin P. Snead

**Affiliations:** From the * Cambridge University Hospitals National Health Service (NHS) Foundation Trust, Cambridge, United Kingdom; † University Division of Anesthesia, Cambridge University Hospitals NHS Foundation Trust, Cambridge, United Kingdom; ‡ Department of Pathology, University of Cambridge, Cambridge, United Kingdom; § Department of Ophthalmology, Vitreoretinal Service, BOX 41, Cambridge University Hospitals NHS Foundation Trust, Cambridge, United Kingdom; ‖ Department of Ophthalmology, Stickler Syndrome Diagnostic Service, Cambridge University Hospitals NHS Foundation Trust, Cambridge, United Kingdom; ¶ Division of Emergency and Perioperative Care, Cambridge University Hospitals NHS Foundation Trust, Cambridge, United Kingdom.

## Abstract

**Background:**

Patients with Stickler syndrome often require emergency surgery and are often anesthetized in nonspecialist units, typically for retinal detachment repair. Despite the occurrence of cleft palate and Pierre-Robin sequence, there is little published literature on airway complications. Our aim was to describe anesthetic practice and complications in a nonselected series of Stickler syndrome cases. To our knowledge, this is the largest such series in the published literature.

**METHODS:**

We retrospectively identified patients with genetically confirmed Stickler syndrome who had undergone general anesthesia in a major teaching hospital, seeking to identify factors that predicted patients who would require more than 1 attempt to correctly site an endotracheal tube (ETT) or supraglottic airway device (SAD). Patient demographics, associated factors, and anesthetic complications were collected. Descriptive statistical analysis and logistic regression modeling were performed.

**RESULTS:**

Five hundred and two

anesthetic events were analyzed. Three hundred ninety-five (92.7%) type 1 Stickler and 63 (96.9%) type 2 Stickler patients could be managed with a single attempt of passing an ETT or SAD. Advanced airway techniques were required on 4 occasions, and we report no major complications. On logistic regression, modeling receding mandible (*P* = .0004) and history of cleft palate (*P* = .0004) were significantly associated with the need for more than 1 attempt at airway manipulation.

**CONCLUSIONS:**

The majority of Stickler patients can be anesthetized safely with standard management. If patients have a receding mandible or history of cleft, an experienced anesthetist familiar with Stickler syndrome should manage the patient. We recommend that patients identified to have a difficult airway wear an alert bracelet.

**KEY POINTS**

- **Question:** Are Stickler syndrome patients at increased risk of major airway complications during anesthesia?
- **Findings:** Of 502 anesthetic events, most patients could be managed with a single airway attempt, 6 patients required advanced airway techniques, and there were no major complications.
- **Meaning:** Few Stickler syndrome patients have a difficult airway requiring advanced airway techniques, whereas the majority of Stickler syndrome patients can be anesthetized safely with standard management.

## SIGNIFICANCE

Stickler syndrome (MIM: 108300, 604841, 614134) is a genetic disorder that most commonly arises from mutations in the genes encoding collagen types II, IX, and XI. First described by Stickler in 1965,^1^ it is estimated to affect between 1 in 7500 and 1 in 9000 newborns.^2^ In addition to other surgical services, patients often require emergency surgery for ophthalmic retinal detachment repair in those patients who have not had prior prophylaxis.^3^ To our knowledge, there are no large published case series about anesthetic complications in Stickler patients, and information is limited to a few published case studies,^4–6^ all of which suggest that the airway is of concern. With a review of cases spanning over 40 years, we present the largest retrospective study of anesthesia for Stickler syndrome in the literature.

### Background

The prevalence of cleft palate in Stickler syndrome patients is estimated at approximately 40%, and 24% of patients are born with Pierre-Robin sequence (PRS).^3^ PRS especially is known to predispose patients to perioperative airway and respiratory concerns.^7^ Further craniofacial manifestations in Stickler syndrome include midface hypoplasia, a small jaw, high-arched palate, and a bifid uvula. An allelic disorder of type 1 Stickler syndrome is spondyloepiphyseal dysplasia congenita (SEDC). Apart from shared phenotypic traits such as myopia and cleft palate,^8^ it is further characterized by skeletal abnormalities such as short stature, kyphoscoliosis, and atlanto-axial instability.^9^ Therefore, these patients may be at risk of cervical spine injury during airway management.^10^

### Rationale

While current literature suggests airway complications are a concern in Stickler syndrome patients, their frequency remains uncertain. This may be due to the rarity of Stickler syndrome, meaning that individual centers will anaesthetize few cases. However, as the national referral center for the provision of surgical services to individuals with Stickler syndrome, our records can provide important messages regarding the frequency of complications. This is particularly important as these patients often require emergency surgery for which transfer to a specialist center may not be possible.

### Objectives

We aimed to describe peri- and postoperative airway complications in individuals with Stickler syndrome undergoing general anesthesia. Our primary end point was major complications, as defined by the fourth national audit project of the Royal College of Anaesthetists and Difficult Airway Society.^11^ Secondary end points were use of advanced airway techniques and failure to secure an airway with a single attempt.

## METHODS

The Cambridge University Hospitals Trust patient safety and audit department approved this retrospective case series as an evaluation of service (reference number: 706) and waived the requirement for written informed consent. The study adheres to Strengthening the Reporting of Observational studies in Epidemiology (STROBE) guidelines.

### Setting

Cambridge University Hospitals National Health Service (NHS) Foundation Trust is a tertiary-level surgical center and teaching hospital located in Cambridge, United Kingdom. The hospital provides the only NHS-commissioned highly specialized service for Stickler syndrome patients in the country. Since October 2014, all medical records have been digitally stored in the electronic health record “EPIC” (Epic systems, Verona, WI). As well as encompassing all aspects of a patient’s medical record, the software also has an integrated Anesthesia Information Management System (AIMS) that records all aspects of an individual’s anesthetic.

### Case Identification

Sample size was limited by available patient records. We searched our electronic health record for anesthetic records of patients with Stickler syndrome who underwent general anesthesia between October 2014 and November 23, 2017. We generated a search algorithm including the following criteria: “Diagnosis”: “Stickler syndrome/Autosomal dominant Stickler syndrome/Nonsyndromic ocular Stickler syndrome/COL11A1-related Stickler syndrome, COL11A2-related Stickler syndrome, COL2A1-related Stickler syndrome” and combinations of the above keywords. Patients must have had an operative encounter. Patients identified by our search were cross-referenced against a prospectively held Stickler service patient database. Data were collected from anesthetic preassessment, intraoperative documentation, and recovery notes if available. We searched patient letters and clinic notes for phenotype, genotype, and patient comorbidities. Based on the Stickler service database, we also hand searched physical medical notes for anesthetic events before October 2014, with the earliest record being from 1971. Patients without phenotypically confirmed Stickler syndrome, available notes, or a record of a general anesthetic were excluded from the data collection.

Patient demographics, operation type, genotype, phenotype, comorbidities, anesthetic risk assessment, and preoperative, intraoperative, and postoperative events were collected for each anesthetic event. The database was anonymized, and all patient identifiable details were removed before further analysis.

### Outcomes

Major complications were defined using the same criteria for major complications used in the fourth national audit project of the Royal College of Anaesthetists and Difficult Airway Society.^11^ These included unplanned intensive treatment unit (ITU) admission or a prolonged ITU stay, brain damage, emergency surgical airway, or death. We defined “advanced airway management” as the use of videolaryngoscopy or fiberoptic intubation devices in either awake or asleep patients. We defined “straightforward airway management” as a single attempt at either inserting an endotracheal tube (ETT) or supraglottic airway device (SAD). Outcomes were defined a priori.

### Statistical Analysis

Basic descriptive statistics were calculated in Microsoft Excel (Microsoft Corp, Redmond, WA). The 95% confidence intervals for proportions were calculated using the Wald or adjusted Wald method.^12^ Missing data were deemed to be missing at random. For the identification of factors associated with an inability to secure the airway at the first attempt, we proceeded to logistic regression analysis. This analysis was performed in R (The R project for statistical computing, https://www.r-project.com). Model generation proceeded as follows: initial univariable analysis of categorical predictors using the χ^2^ test (or Fisher exact test where cell frequencies were ≤5) was performed. Variables associated with a *P* value of <.1 were considered for progression to multivariable modeling. These variables were introduced into a regression model against our outcome of interest. Model simplification was performed with backward step regression. Variables were excluded if the nested, simplified model was not significantly different (*P* > .05) from the original model on likelihood ratio testing.

We performed a sensitivity analysis to look for any residual confounding by variables that we did not carry forward to our model building process. For this, we built 2 additional multivariable models, including additional variables with *P* > .1 on initial testing in “airway and anesthetic characteristics” and “specialty.” These models were compared to the nested model formed from our stepwise regression using likelihood ratio testing.

## RESULTS

### Patient Characteristics

We initially collected 1004 anesthetic events from 540 patients. We only included the first anesthetic event of each patient to prevent pseudo-replication and to avoid linkage error. We excluded patients without a primary outcome and those with a diagnosis other than Stickler syndrome type 1, type 2, and SEDC according to published criteria.^13^ This left us with 502 patients for analysis.

Four hundred eleven patients (81.9%) had a personalized genetic confirmation. Four hundred twenty-six were diagnosed with type 1 Stickler syndrome, 65 as type 2 Stickler syndrome, and 11 with SEDC. There were 482 anesthetic events for ophthalmological procedures and 20 for other surgical specialties, including orthopedics (7 cases); ear, nose, and throat (4 cases); plastic surgery (4 cases); general surgery (3 cases); neurosurgery (1 case); and transplant (1 case).

**Table 1.** Phenotypic Traits of Patients by Stickler Syndrome Phenotype and Patient Comorbidities

	Type 1 Stickler Syndrome (n = 426), n (%)	Type 2 Stickler Syndrome (n = 65), n (%)	SEDC (n = 11), n (%)
Phenotypic traits			
Cataract	74 (17.4)	15 (23.1)	3 (27.3)
Glaucoma	12 (2.8)	4 (6.2)	0 (0)
Retina	188 (44.1)	30 (46.2)	4 (36.4)
Joint involvement	153 (35.9)	22 (33.8)	4 (36.4)
Scoliosis	2 (0.5)	1 (1.5)	1 (9.1)
Cervical spine	0 (0)	0 (0)	1 (9.1)
Hearing	101 (23.7)	24 (36.9)	0 (0)
Previous cleft repair	165 (38.7)	16 (24.6)	3 (27.3)
Pierre-Robin sequence	58 (13.6)	1 (1.5)	0 (0)
Bifid uvula	8 (1.9)	1 (1.5)	0 (0)
High-arched palate	20 (4.7)	1 (1.5)	0 (0)
Comorbidities			
Hypertension	16 (3.8)	4 (6.2)	0 (0)
Other cardiac	14 (3.3)	2 (3.1)	0 (0)
Reflux	11 (2.6)	2 (3.1)	0 (0)
Asthma/COPD	47 (11.0)	9 (13.8)	3 (27.3)
Smoking	3 (0.7)	0 (0)	0 (0)
Atopy	4 (0.9)	1 (1.5)	0 (0)
Diabetes mellitus	3 (0.7)	0 (0)	0 (0)
Other endocrine	5 (1.2)	2 (3.1)	0 (0)
Prematurity	2 (0.5)	0 (0)	0 (0)
Epilepsy	4 (0.9)	0 (0)	0 (0)
Concurrent syndrome	2 (0.5)	1 (1.5)	0 (0)

Abbreviations: COPD, chronic obstructive pulmonary disease; SEDC, spondyloepiphyseal dysplasia congenita.

**Figure.**
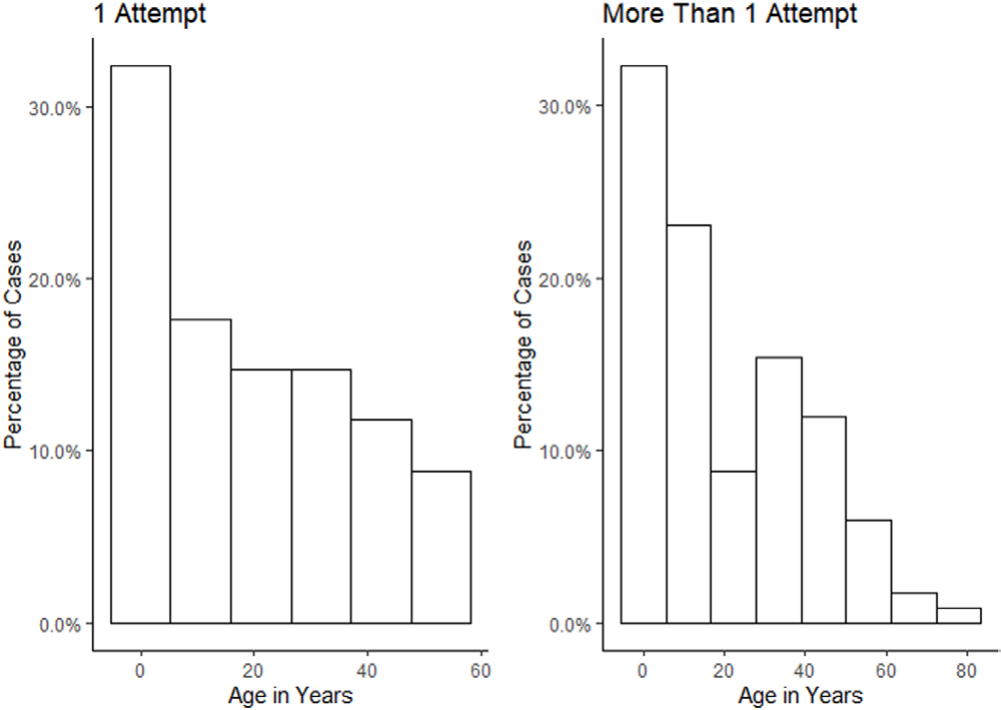
Age distribution of patients with a successful and unsuccessful first intubation attempt. *P* = .70.

Median age was 12 (interquartile range [IQR], 4–37) for type 1 Stickler syndrome patients, 10 (IQR 4–35) for type 2 Stickler syndrome patients, and 18 (IQR 7–39) for SEDC patients. Two hundred thirty-seven type 1 Stickler syndrome patients (55.6% of type 1 Stickler patients) were <18 years of age, 42 (64.6%) type 2 Stickler syndrome patients, and 5 (45.5%) SEDC patients. Body mass index (BMI) was recorded in 62 cases of the adult (over 18 years old) patient sample. The mean BMI was 28 (standard deviation = 6.7). Phenotypic variation within the patient group and patient comorbidities are summarized in Table 1. Age distribution of our cohort is demonstrated in the Figure separated by the presence or absence of airway complications.

### Surgical Procedures

According to the National Confidential Enquiry into Patient Outcome and Death (NCEPOD) classification, 484 procedures were elective, 6 were classed as urgent, 3 as expedited, 5 as emergency, and 4 were not classified.

**Table 2.** Characteristics of Patients Relevant to the Anesthetic Risk Assessment for Each Stickler Syndrome Phenotype

	Type 1 Stickler Syndrome (n = 426), n (%)	Type 2 Stickler Syndrome (n = 65), n (%)	SEDC (n = 11), n (%)
Airway characteristics			
Poor mouth opening	8 (1.9)	4 (6.2)	0 (0)
Micrognathia	30 (7.0)	2 (3.1)	0 (0)
Snoring	6 (1.4)	0 (0)	0 (0)
Obstructive sleep apnea	7 (1.6)	1 (0)	0 (0)
Mallampati score			
I	95 (22.3)	11 (16.9)	2 (18.2)
II	67 (15.7)	14 (21.5)	1 (9.1)
III	11 (2.6)	4 (6.2)	0 (0)
IV	0 (0)	1 (1.5)	0 (0)
No data	253 (59.4)	35 (53.8)	8 (72.7)
ASA physical status			
I	141 (33.1)	27 (41.5)	3 (27.3)
II	207 (48.6)	26 (40)	7 (63.6)
III	17 (4.0)	5 (7.7)	1 (9.1)
No data	61 (14.3)	7 (10.8)	0 (0)
Cormack Lehane grade			
1	36 (8.5)	7 (10.8)	1 (9.1)
2	11 (2.6)	2 (3.1)	0 (0)
3	6 (1.4)	2 (3.1)	1 (9.1)
4	0 (0)	0 (0)	0 (0)
No data	373 (87.6)	54 (83.1)	9 (81.8)

Abbreviations: ASA, American Society of Anesthesiologists physical status classification system; SEDC, spondyloepiphyseal dysplasia congenita.

Three hundred fifty patients (69.7%) had a history of surgery in another hospital. Documented anesthetic complications in other hospitals included postoperative nausea and vomiting (PONV) (20 patients), difficult airway (6 patients), anxiety (1 patient), headache (1 patient), torticollis (1 patient), subglottic stenosis (1 patient), and breathing difficulty postoperatively (1 patient). Anesthetic risk assessment is summarized in Table 2.

### Perioperative Findings

Reassuringly, there were no documented cases of “can’t intubate, can’t oxygenate.” There were no abandoned procedures because of failed airway management, no major complications, or emergency front of neck airway.

Advanced airway techniques were used on 6 occasions (1.19%). One patient had SEDC, and all others were type 1 Stickler syndrome patients. Fiberoptic intubation was used twice, and Airtraq was used 4 times.

Airway management is summarized in Table 3. Overall, the airway of 468 patients (93.2%) was successfully managed by a single attempt. Thirty-four patients (6.8%, 95% confidence interval, 4.6–9.0) required multiple attempts. Broken down into subtypes, 31 (7.3%, 95% confidence interval, 4.8–9.7) type 1 Stickler syndrome patients did not have a successful single attempt at managing the airway, 2 (3.1%, 95% confidence interval, 0.2–11.17) type 2 patients and no SEDC (0%, 95% Confidence Interval 0–23.1) patients required multiple airway attempts. Six of these first-time successful attempts were documented as difficult in type 1 Stickler syndrome patients. Likewise, 1 type 2 Stickler syndrome patient had a successful first attempt at intubation that was documented as difficult.

**Table 3.** Airway Management for Each Stickler Syndrome Phenotype

	Type 1 Stickler (n = 426), n (%)	Type 2 Stickler (n = 65), n (%)	SEDC (n = 11), n (%)
SAD, single attempt	327 (76.8)	51 (78.5)	8 (72.7)
Endotracheal tube, single attempt	68 (16.0)	12 (18.5)	2 (18.2)
Multiple attempts	17 (4.0)	1 (1.5)	0 (0)
Conversion to different device	9 (2.1)	1 (1.5)	0 (0)
Advanced airway technique	5 (1.2)	0 (0)	1 (9.1)

Conversion to a different device excluded patients who required advanced airway techniques.

Abbreviations: SAD, supraglottic airway device; SEDC, spondyloepiphyseal dysplasia congenita.

Patients required laryngeal manipulation on 8 occasions and a bougie or stylet in 3 cases. Additional guidance for inserting an SAD using a laryngoscope was required on 1 occasion. The seal of 9 SADs was documented as poor. It was accepted in 2 cases, required a change of size in 2 cases, and a change to ETT in 5 cases. Seal of an ETT was poor but acceptable in 2 cases.

All patients were able to be ventilated using bag and mask ventilation, although 3 (0.70%) type 1 Stickler patients required 2-handed ventilation to achieve this.

Inhalational induction of anesthesia was performed in 137 cases (27.3%), otherwise an intravenous induction was used. SEDC patients were held in in-line stabilization due to their cervical spine instability where this was a clinical concern. On extubation, there was 1 case of pneumothorax after central line insertion during a neurosurgical procedure (resection of acoustic neuroma), requiring a chest drain.

### Logistic Regression Analysis

**Table 4.** Univariate Analysis of Associated Factors for an Airway That Could Not Be Secured at the First Attempt

	Unable to Secure First Time (n = 34), n (%) or Median [IQR]	Able to Secure First Time (n = 468), n (%) or Median [IQR]	*P*
Stickler phenotypes			
Type 1 phenotype^a^	31 (91.2)	395 (84.4)	.29
Type 2 phenotype^b^	2 (5.9)	63 (13.5)	.29
SEDC phenotype^b^	1 (2.9)	10 (2.1)	.54
Phenotype characteristics			
Cataract^b^	4 (11.8)	88 (18.8)	.37
Glaucoma^b^	2 (5.9)	14 (3.0)	.30
Retinal pathology^a^	13 (38.2)	209 (44.7)	.47
Joint involvement^a^	10 (29.4)	169 (36.1)	.43
Scoliosis^b^	0 (0)	4 (0.9)	1
Cervical spine instability^b^	0 (0)	1 (0.2)	1
Hearing impairment^a^	8 (23.5)	117 (25.0)	.85
Airway and anesthetic characteristics			
Pierre-Robin syndrome^b^	5 (14.7)	54 (11.5)	.58
Bifid uvula^b^	0 (0)	9 (1.9)	1
High-arched palate^b^	2 (5.9)	19 (4.1)	.65
Previous cleft repair^a^	22 (64.7)	162 (34.6)	**.0004**
History of difficult intubation^b^	0 (0)	6 (1.3)	1
Mallampati 3+^b^	1 (2.9)	16 (3.4)	1
Mouth opening >3 fingers^a^	33 (97.1)	457 (97.6)	.83
Receding jaw^a^	7 (20.6)	25 (5.3)	**.0004**
Obstructive sleep apnea^b^	1 (2.9)	7 (1.5)	.43
Demographics			
Age^c^	14 [2.25–35.75]	14 [4–38]	.7
Specialty			
Ophthalmic surgery^a^	31 (91.2)	451 (96.4)	.14

Statistical tests were chosen as appropriate for each variable. Bold text indicates *P* < .05.

Abbreviations: IQR, interquartile range; SEDC, spondyloepiphyseal dysplasia congenita.

^a^χ^2^ test.

^b^Fisher exact test.

^c^Mann-Whitney *U* test.

**Table 5.** Adjusted Odds Ratios and Confidence Intervals for the Independent Variables of the Final Model

	Odds Ratio (95% Confidence Interval)
Previous cleft repair	2.9 (1.4–6.3)
Receding jaw	3.0 (1.1–7.5)

At the prespecified significance level, 2 factors (receding jaw and cleft) were significant in univariable testing (Table 4; Figure). On multivariable testing, the initial model consisting of these 2 variables did not simplify further, with a significant likelihood ratio test between the 2 models (*P* = .04). The model has poor discriminative capability, with an area under the receiver operator curve of 0.68 (95% confidence interval, 0.59–0.77). Odds ratios are reported in Table 5. Sensitivity analysis models are shown in Supplemental Digital Content, Tables 1–2, http://links.lww.com/AA/C977. When a full range of airway characteristics were included, the odds ratio for our 2 final variables shifted by 16%, suggesting a degree of residual confounding although no change in overall conclusions. However, models showed no statistically significant difference in likelihood ratio testing (*P* = .78 for airway and anesthetic characteristics and *P* = .18 for specialty versus stepwise model).

### Postoperative Complications

All patients received care in the postanesthesia care unit in accordance with unit protocols at the time. This included as-needed prescriptions for analgesia and antiemetics. Fifteen patients (3.0%) required manual airway assistance in the form of a chin lift or jaw thrust. Fifty patients (10.0%) had documented PONV.

## DISCUSSION

### Principal Findings

In this, the largest series examining airway management in Stickler syndrome patients, it is reassuring that we did not identify any major airway complications or requirement for emergency front-of-neck access. Furthermore, over 90% of all patients with Stickler syndrome had a documented first-time success with the chosen airway strategy (either insertion of a SAD or insertion of an ETT). This is reassuring, as this should represent a very sensitive indicator of airway difficulty. Our findings show that a cleft palate and a receding mandible are associated with multiple attempts at managing the airway. The model could not be simplified further, indicating that the patients identified by the 2 variables are different. Although the independent variables were significant in univariate testing, the discriminative capacity of our final model is poor, with an area under the receiver operator curve of 0.68.

Within our cohort, 350 patients had procedures performed in other hospitals. Our findings may thus be useful in providing guidance for anesthetists unfamiliar with the genetic condition.

### Strengths and Weaknesses

We acknowledge that our study is not without its limitations. First, as a retrospective, single-center case series, it has no external validity. A major limitation stems from our use of routinely collected data for epidemiological analysis. It is likely that our results are influenced by documentation errors or omissions. Individual anesthetists may have differing opinions in terms of what should be recorded, introducing the opportunity for marked interrater variability. One could hypothesize that cases where serious complications (eg, failed airway) occurred, might have a higher degree of completion than simple changes in techniques (eg, need for 2-handed ventilation). Furthermore, it is possible that any misclassification resulting from the recorded documentation may be differential between those with a “difficult” airway versus an uneventful airway. If this were the case, then this introduces information bias pertaining to our classification of specific events between our groups of interests. Further variation between recording could have occurred due to our inclusion of individuals whose records were either paper based or hosted within our electronic health record. Our electronic health record mandates the recording of certain types of information before allowing a record to be closed.

There is also the potential for significant residual confounding. We did not collect information on the grade or experience level of the anesthetist. As operating lists are often managed by the same anesthetist, there is likely a degree of operator dependency. Anesthetists with more experience in anesthetizing Stickler syndrome patients could have higher rates of successful airway management or be less likely to utilize advanced techniques (eg, fiberoptic intubation) as a routine. Other confounding factors that were not accounted for could include equipment availability or the teaching of anesthetic trainees.

### Comparison to Other Studies

Comparison of airway complications between studies is difficult due to the lack of a consensus definition for a difficult airway in the literature.^14^ Although our primary outcome was the occurrence of major airway complications,^11^ we also defined successful airway management as consisting of a single attempt using either an SAD or ETT. In certain settings (eg, emergency gastrointestinal surgery), the use of an SAD may not be an appropriate airway management strategy. It is conceivable that certain individuals might be a difficult intubation but are easily managed using an SAD. As such, our pooled outcome may be an underestimate of airway difficulty in the setting of a more diverse range of surgical procedures. Regardless, the reliability of SAD insertion in these patients should be reassuring if intubation difficulty was encountered in other settings.

A Danish cohort study found that the overall proportion of patients who underwent difficult tracheal intubation was 5.1%, defined as intubated by the first anesthetist in more than 2 attempts.^15^ Failed intubation (defined as change of method or abandoned procedure) occurred in 1.9% of cases.

It is unsurprising that anesthetic risk factors were not associated with more than 1 attempt at managing the airway in our Stickler syndrome patient population. It is well described that the diagnostic accuracy of predicting a difficult airway is low. A Danish cohort study found that 93% of difficult airways were unanticipated, while only 25% of anticipated difficult airways were indeed difficult.^16^ A recent Cochrane meta-analysis confirmed that bedside tests such as the Modified Mallampati test have a low sensitivity.^17^ In addition, literature on anesthetic risk factors mention BMI, snoring, and sleep apnea as possible predictors of difficult intubation and ventilation.^18–20^ In our case series, we could not confirm these as associated factors.

Both cleft and a receding mandible are clinical features of PRS.^21^ These patients are known to have a difficult airway, especially as neonates.^7^ Nevertheless, PRS was not identified as an associated factor by our case series. This may be due to the age of the patient at the time of surgery. PRS patients may experience mandibular catch-up growth, as airway anatomy is known to improve with age.^22^ For our patients, a medical history of PRS may be present, but the clinical features may have changed with time.

Postoperatively, we found that 3% of patients had postoperative respiratory complications requiring manual airway maneuvers. We did not find any major postoperative complications, and also in the general population significant events such as death or hypoxic brain injury are rare.^23^ A UK audit based in a district general hospital reports 2.8% airway problems,^24^ and an Australian study of 13,266 anesthetic events found that anesthetist intervention for respiratory problems was necessary in 1.0%–1.5% of postoperative patients.^25^ Our findings, especially for type 1 Stickler syndrome patients, seem to be slightly higher than the reported incidence in the general population. By contrast to other studies, we have included complications that were easily managed by recovery room nursing staff and that did not require anesthetist intervention, which may account for the higher incidence.

## CONCLUSIONS

Our case series found that anesthesia in Stickler syndrome patients is associated with low rates of airway complications. Both endotracheal and SAD techniques are successful methods of securing the airway. Difficulty and failure of securing the airway may be similar to the general population, bearing in mind the limitations of the study and lack of external validity. Patients with a receding jaw or a history of cleft palate may represent a higher risk group and should be managed by a senior anesthetist experienced in the management of patients with Stickler syndrome. In light of our findings, we also recommend that Stickler syndrome patients with a known difficult airway wear an alert bracelet.

## DISCLOSURES

**Name:** Julia Zimmermann, MBBS.

**Contribution:** This author helped with design of the analysis, data acquisition and statistical analysis, and preparing and revising the manuscript.

**Name:** Daniel J. Stubbs, BM BCh, MA.

**Contribution:** This author helped with statistical methodology, statistical analysis, and drafting the manuscript.

**Name:** Allan J. Richards, PhD.

**Contribution:** This author helped with conception and drafting of the manuscript.

**Name:** Philip Alexander, MBBS, DM.

**Contribution:** This author helped with conception and drafting of the manuscript.

**Name:** Annie M. McNinch, SRN.

**Contribution:** This author helped with conception and data acquisition.

**Name:** Basil Matta, BCh MB, DA.

**Contribution:** This author helped with conception and design of the analysis, data acquisition, and preparing the manuscript.

**Name:** Martin P. Snead, MD.

**Contribution:** This author helped with conception and design of the analysis, data acquisition, and preparing the manuscript.

**This manuscript was handled by:** David Hillman, MD.

## Supplementary Material


