# Supplementary material for: Stickler Syndrome: Airway Complications in a Case Series of 502 Patients
Source: Anesth Analg. 2019 Dec 16;132(1):202–9. doi: 10.1213/ANE.0000000000004582 (PMC7717475; doi:10.1213/ANE.0000000000004582)
Supplement: Supplementary file 1 [file ane-132-202-s001.pdf]

Supplementary material Table 1: Odds ratio, confidence interval and p-values for Sensitivity analysis model 1: Airway and anesthetic characteristics

	Odds ratio (95% Confidence Interval)	p-value
Pierre-Robin sequence	0.6 (0.2-1.5)	0.28
High arched palate	1.4 (0.2-5.8)	0.67
Previous Cleft repair	3.4 (1.5-7.6)	0.003
Mouth opening >3 Fingers	1.3 (0.2-28.2)	0.81
Receding Jaw	3.2 (1.1-8.5)	0.02
Obstructive Sleep Apnoea	1.4 (0.1-9.3)	0.77

Supplementary material Table 2: Odds ratio, confidence interval and p-values for Sensitivity analysis model 2: Specialty

	Odds ratio (95% Confidence Interval)	p-value
Previous Cleft repair	2.9 (1.4-6.3)	0.007
Receding Jaw	3.0 (1.1-7.8)	0.03
Ophthalmic surgery	0.4 (0.1-1.7)	0.15
